# A224 BARRIERS AND FACILITATORS TO IMPLEMENTATION OF A NURSE NAVIGATOR-LED, EVIDENCE-BASED, IBD FLARE PATHWAY; RESULTS FROM A NATIONAL MULTISTAKEHOLDER ENGAGEMENT PROCESS

**DOI:** 10.1093/jcag/gwae059.224

**Published:** 2025-02-10

**Authors:** N Willett, C Heisler, J Marshall, M Stewart, L Jimenez, A Bennett, J Robar, B Currie, K Phalen-Kelly, J Jones

**Affiliations:** Division of Digestive Care and Endoscopy, Nova Scotia Health Authority, Halifax, NS, Canada; Division of Digestive Care and Endoscopy, Nova Scotia Health Authority, Halifax, NS, Canada; McMaster University, Hamilton, ON, Canada; Division of Digestive Care and Endoscopy, Nova Scotia Health Authority, Halifax, NS, Canada; Dalhousie University, Halifax, NS, Canada; Division of Digestive Care and Endoscopy, Nova Scotia Health Authority, Halifax, NS, Canada; Division of Digestive Care and Endoscopy, Nova Scotia Health Authority, Halifax, NS, Canada; Division of Digestive Care and Endoscopy, Nova Scotia Health Authority, Halifax, NS, Canada; Division of Digestive Care and Endoscopy, Nova Scotia Health Authority, Halifax, NS, Canada; Division of Digestive Care and Endoscopy, Nova Scotia Health Authority, Halifax, NS, Canada

## Abstract

**Background:**

The clinical resources of many IBD programs are insufficient to meet population and care complexity needs for persons living with IBD. Implementation of evidence-based pathways via an existing provincial nurse navigator could improve access to evidence-based care for persons living with IBD. Stakeholder engagement is important when identifying barriers to implementation and adoption of evidence-based interventions. Groups interviews were performed as part of a national community advisory panel with nurses and gastroenterologists to better understand barriers and facilitators to implementation of evidence-based care guidelines for IBD flares.

**Aims:**

To identify barriers and facilitators to implementation of evidence based IBD flare pathways in clinical practice through a nurse-led initiative.

**Methods:**

Two semi-structured group interviews were performed as part of the national PACE CCC advisory panel in September 2022. Group interviews were via Zoom, each 1 hour in length. Interview script questions were guided by the COM-B implementation science framework. GI interviews was facilitated by a gastroenterologist (JM) and the nursing group interviews by a research assistant (CH). Results were derived from thematic analysis.

**Results:**

A total of 16 IBD care providers participated. One group represented nurses (n=8), while the other included gastroenterologists (n=6) and members of Crohn’s and Colitis Canada (n=2). Thematic barriers to creating and implementing a flare pathway were identified as: Access to care issues, resources and staffing issues, COVID-19 impact, poor communication between specialists and community-based providers, variability of local resources (imaging, specialists, endoscopy), and cost of implementation. Facilitators included: Virtual care platforms, standardization of care, multidisciplinary teams, patient empowerment, leveraging digital health tools for customisation and implementation of the pathway (i.e., EMR templates). Intervention functions included: Empowering nurse navigators to manage flare assessments and improving access to diagnostic testing and resources by embedding pathways into electronic health records.

**Conclusions:**

Nurses and Gastroenterologists shared invaluable insights into the status of IBD Care in Canada and ways to improve access to evidence-based, and equitable IBD care during a flare. Overall, evidence- based care pathways were viewed as being of importance and that implementation strategies should focus on leveraging digital health platforms, EMRs, and empower IBD nurses to facilitate IBD flare pathway implementation.

**Funding Agencies:**

CCC

